# Development and Validation of a Nomogram to Predict Cancer-Specific Survival in Elderly Patients With Papillary Renal Cell Carcinoma

**DOI:** 10.3389/fpubh.2022.874427

**Published:** 2022-04-04

**Authors:** Chenghao Zhanghuang, Jinkui Wang, Zhigang Yao, Li Li, Yucheng Xie, Haoyu Tang, Kun Zhang, Chengchuang Wu, Zhen Yang, Bing Yan

**Affiliations:** ^1^Department of Urology, Kunming Children's Hospital (Children's Hospital Affiliated to Kunming Medical University), Kunming, China; ^2^Department of Urology, Chongqing Key Laboratory of Children Urogenital Development and Tissue Engineering, Chongqing Key Laboratory of Pediatrics, Ministry of Education Key Laboratory of Child Development and Disorders, National Clinical Research Center for Child Health and Disorders, China International Science and Technology Cooperation Base of Child Development and Critical Disorders, Children's Hospital of Chongqing Medical University, Chongqing, China; ^3^Yunnan Key Laboratory of Children's Major Disease Research, Kunming Children's Hospital (Children's Hospital Affiliated to Kunming Medical University), Kunming, China; ^4^Department of Pathology, Kunming Children's Hospital (Children's Hospital Affiliated to Kunming Medical University), Kunming, China; ^5^Department of Oncology, Yunnan Children Solid Tumor Treatment Center, Kunming Children's Hospital (Children's Hospital Affiliated to Kunming Medical University), Kunming, China

**Keywords:** nomogram, papillary renal cell carcinoma, cancer-specific survival, elderly patients, SEER

## Abstract

**Objective:**

Papillary renal cell carcinoma (pRCC) is the second most common type of renal cell carcinoma and an important disease affecting older patients. We aimed to establish a nomogram to predict cancer-specific survival (CSS) in elderly patients with pRCC.

**Methods:**

Patient information was downloaded from the Surveillance, Epidemiology, and End Results (SEER) project, and we included all elderly patients with pRCC from 2004 to 2018. All patients were randomly divided into a training cohort and a validation cohort. Univariate and multivariate Cox proportional risk regression models were used to identify patient independent risk factors. We constructed a nomogram based on a multivariate Cox regression model to predict CSS for 1-, 3-, and 5- years in elderly patients with pRCC. A series of validation methods were used to validate the accuracy and reliability of the model, including consistency index (C-index), calibration curve, and area under the Subject operating curve (AUC).

**Results:**

A total of 13,105 elderly patients with pRCC were enrolled. Univariate and multivariate Cox regression analysis suggested that age, tumor size, histological grade, TNM stage, surgery, radiotherapy and chemotherapy were independent risk factors for survival. We constructed a nomogram to predict patients' CSS. The training and validation cohort's C-index were 0.853 (95%CI: 0.859–0.847) and 0.855 (95%CI: 0.865–0.845), respectively, suggesting that the model had good discrimination ability. The AUC showed the same results. The calibration curve also indicates that the model has good accuracy.

**Conclusions:**

In this study, we constructed a nomogram to predict the CSS of elderly pRCC patients, which has good accuracy and reliability and can help doctors and patients make clinical decisions.

## Background

Renal cell carcinoma (RCC) is the most common Renal malignant tumor in adults, accounting for 90% of renal tumors (1). RCC is divided into three main types based on histological features, with papillary renal cell carcinoma (pRCC) being the second most common type, accounting for ~10 to 15% of the total number of diseases. Clear cell renal cell carcinoma (ccRCC) accounts for 70–80% of these cases, and chromophobe renal cell carcinoma (cRCC) remains in the rest (2, 3). According to pathological features, pRCC is divided into two main subtypes: Type I papillary renal cell carcinoma is characterized by unique basophilic papillary cells. In contrast, Type II is characterized by many papillary cells, and the cytoplasm of type II pRCC is eosinophilic (4). It is worth noting that compared with other RCC, pRCC has special clinical manifestations, biological behaviors and pathological morphology, and its diagnosis and treatment are also different from other RCCS, which are still controversial (5, 6).

Around the world, 400,000 people are diagnosed with RCC every year (1), and the elderly over 60 years old account for more than 75% of the cases (7). In addition, with the aggravation of population aging and the extension of life expectancy, the incidence rate of renal cancer in the elderly is also increasing year by year (8). At present, the prognosis of pRCC is still poor, especially for advanced patients, and there is no effective treatment (9). Therefore, it is particularly important to judge the prognosis of elderly pRCC patients accurately.

Traditionally, TNM staging has been regarded as the main criteria for the prognosis of various malignant tumors. However, it is not enough to cover the biological characteristics of various malignant tumors nor to validate the survival outcome (10). Other clinical variables, such as age, sex, race, grade, surgical treatment, adjuvant therapy, and molecular characteristics, may also impact the outcome of cancer patients.

In recent years, the nomogram prediction model, including UISS (11), SSIGN (12), etc., is considered to be one of the most accurate methods for tumor prediction (13). However, there are no relevant reports of these clinical variables on elderly pRCC cases at the present stage (14). The objective of this retrospective study was to investigate the clinicopathological features associated with the prognosis of elderly pRCC patients collected from the Surveillance, Epidemiology, and End Results (SEER) database of the National Cancer Institute. We then used these features to construct a nomogram to predict cancer-specific survival of patients with pRCC.

## Patients and Methods

### Data Source and Data Extraction

We downloaded clinicopathological information of all patients with pRCC from 2004 to 2018 to the SEER database. SEER data is the national cancer database of the United States, consisting of 18 cancer registries covering ~30% of the national population. Clinicopathological information and follow-up data for all cancer patients are publicly available from the SEER database. Patient personal information is not identifiable, and SEER database information is publicly available, so we do not need to obtain ethical approval and informed consent from patients. Our research methods strictly follow the rules of SEER data.

**Figure 1:**
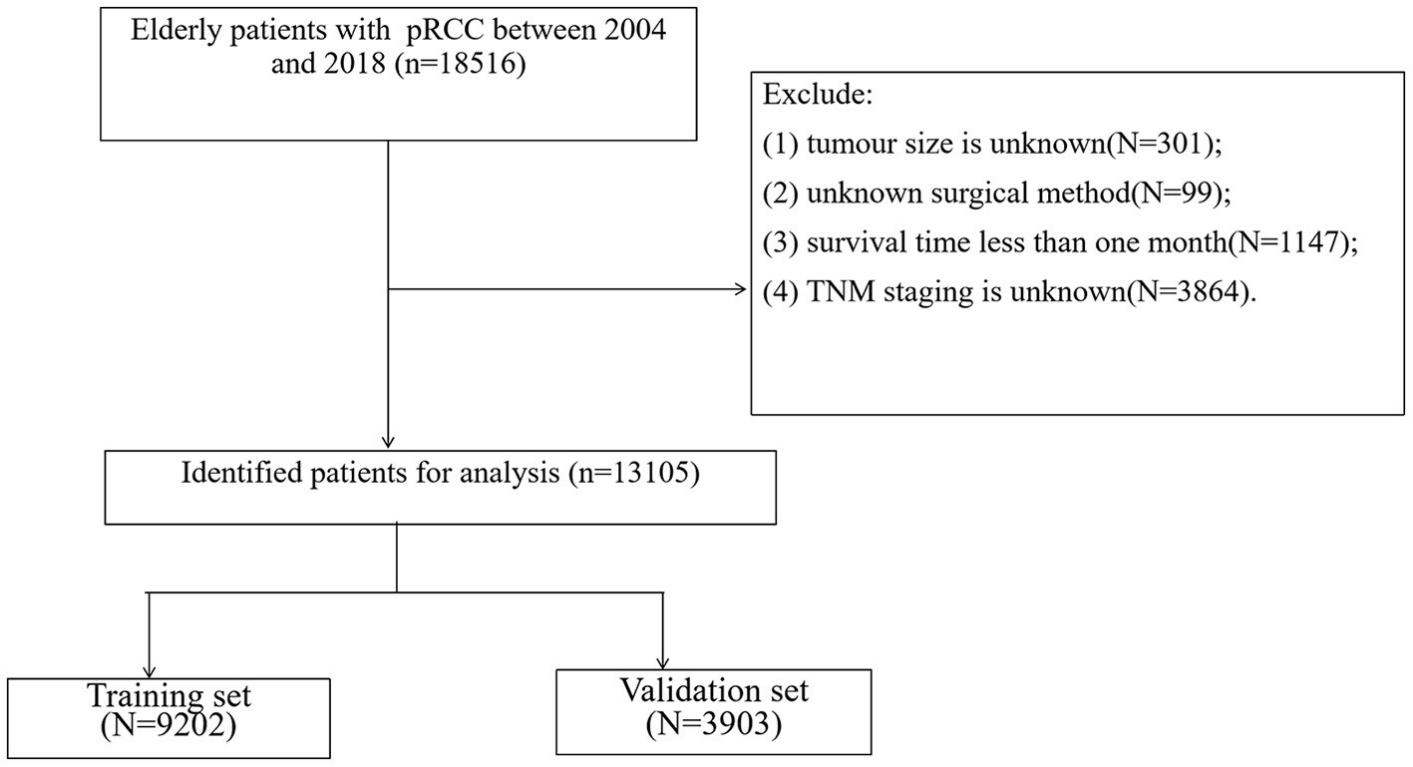
Flowchart for inclusion and exclusion of all patients.

We collected the basic information of the patient, including age, gender, race, year of diagnosis, marital status; we collected the patient's clinical-pathological information, including the tumor size, laterality, histological grade, TNM staging, surgery, radiation therapy, chemotherapy, patients with follow-up information including living status, the cause of death and survival time. Inclusion criteria:(1) pathological diagnosis of papillary renal cell carcinoma (ICD-O-3 code, 8260); (2) Age ≥65; (3) Unilateral renal tumor. Exclusion criteria:(1) TNM staging is unknown; (2) Tumor size is unknown; (3) Unknown surgical method; (4) Survival time <1 month. The screening flow chart of all patients is shown in Figure 1.

The patients' marital status was divided into married and unmarried (single, divorced, widowed); Patients' races were divided into white, black, and others (American Indian /AK Native, Asian/Pacific Islander). The years of diagnosis were divided into between 2004 and 2010 and between 2011 and 2018. The histological grades of the patients included grade I (well differentiated), grade II (moderately differentiated), grade III (poorly differentiated), and grade IV (undifferentiated). The surgical classification of patients included non-surgical (surgical code 0), local tumor resection (surgical code 10–27), partial nephrectomy (surgical code 30), and radical nephrectomy (surgical code 40–80).

### Nomogram Development and Validation

All patients enrolled were randomly assigned to a training cohort (70%) or a validation cohort (30%). In the training cohort, we used a univariate Cox regression model to pre-screen the influencing factors of patients' prognoses. We then used a multivariate Cox proportional risk regression model to determine the independent risk factors for CSS in patients. Based on a multivariate Cox proportional risk regression model, we constructed a new nomogram to predict CSS at 1-, 3-, and 5 years in patients with pRCC. Then, we use a series of validation methods to test the accuracy and discrimination of the prediction model. We used consistency index (C-index) and area under the receiver operating curve (AUC) to test the model's discrimination. Calibration curves of 1,000 bootstrap samples were used to validate the model's accuracy.

### Clinical Utility

A decision analysis curve (DCA) is a new algorithm to calculate the net benefits of models under different thresholds. DCA was used to validate the clinical utility of the nomogram. In addition, we calculated the value of risk for each patient based on the nomogram and used truncation values to divide all patients into high-risk and low-risk groups. Kaplan-Meier (K-M) curves and log-rank tests were used to determine differences in survival among groups.

### Statistical Analysis

Continuous variables (age, tumor size) were described by means and variance, and comparisons between groups were performed by chi-square or non-parametric *U*-tests. Count data were expressed by frequency (%), and a chi-square test was used to compare groups. Univariate and multivariate Cox proportional regression models analyzed the survival and prognostic factors. All statistical analyses were conducted by SPSS 26.0 and R software 4.1.0. A P value <0.05 was considered statistically significant.

## Results

### Clinical Features

Based on inclusion and exclusion criteria, a total of 13,105 elderly patients with pRCC were included. All patients were divided into a training cohort (*N* = 9250) and a validation cohort (*N* = 3855). The mean age of the patients was 75.2 ± 7.57 years, and there were 10936 (83.4%) white patients, 7594 (57.9%) male patients, and 7089 (54.1%) married patients. There were 768 (5.86%) patients at grade I, 2560 (19.5%) at grade II, 1685 (12.9%) at grade III, and 497 (3.79%) at grade IV. There were 5794 (65.8%) patients with stage T1a, 11983 (91.4%) patients with stage N0, and 10665 (81.4%) patients with stage M0. Local tumor excision, partial nephrectomy and radical nephrectomy were performed in 1269 (9.68%), 1519 (11.6%), and 4521 (34.5%) patients, respectively. 1,085 (8.28%) patients underwent chemotherapy, and 638 (4.87%) patients underwent radiotherapy. The clinicopathological information of all patients was shown in Table 1, and there was no significant difference between the training and validation cohorts.

**Table 1:** Clinicopathological characteristics of elderly patients with pRCC.

	**All**	**Training cohort**	**Validation cohort**	
	***N*** **=** **13105**	***N*** **=** **9,202**	***N*** **=** **3,903**	***p***
Age				0.024
65–74	6,847 (52.2%)	4,762 (51.7%)	2,085 (53.4%)	
75–84	4,432 (33.8%)	3,110 (33.8%)	1,322 (33.9%)	
≥85	1,826 (13.9%)	1,330 (14.5%)	496 (12.7%)	
Race				0.404
White	10,936 (83.4%)	7,658 (83.2%)	3,278 (84.0%)	
Black	1,444 (11.0%)	1,036 (11.3%)	408 (10.5%)	
Other	725 (5.53%)	508 (5.52%)	217 (5.56%)	
Sex				0.337
Male	7,594 (57.9%)	5,307 (57.7%)	2,287 (58.6%)	
Female	5,511 (42.1%)	3,895 (42.3%)	1,616 (41.4%)	
Marital				0.002
Married	7,088 (54.1%)	4,885 (53.1%)	2,203 (56.4%)	
Unmarried or Domestic Partner/Single	1,874 (14.3%)	1,341 (14.6%)	533 (13.7%)	
Separated/Divorced/ Widowed	4,143 (31.6%)	2,976 (32.3%)	1,167 (29.9%)	
Year of diagnosis				0.683
2004–2010	6,125 (46.7%)	4,312 (46.9%)	1,813 (46.5%)	
2010–2018	6,980 (53.3%)	4,890 (53.1%)	2,090 (53.5%)	
Laterality				0.660
Left	6,440 (49.1%)	4,510 (49.0%)	1,930 (49.4%)	
Right	6,665 (50.9%)	4,692 (51.0%)	1,973 (50.6%)	
Grade				0.652
I	768 (5.86%)	531 (5.77%)	237 (6.07%)	
II	2,560 (19.5%)	1,785 (19.4%)	775 (19.9%)	
III	1,685 (12.9%)	1,167 (12.7%)	518 (13.3%)	
IV	497 (3.79%)	347 (3.77%)	150 (3.84%)	
Unknown	7,595 (58.0%)	5,372 (58.4%)	2,223 (57.0%)	
T				0.925
T1a	5,794 (44.2%)	4,070 (44.2%)	1,724 (44.2%)	
T1b	3,011 (23.0%)	2,121 (23.0%)	890 (22.8%)	
T2	1,606 (12.3%)	1,137 (12.4%)	469 (12.0%)	
T3	2,607 (19.9%)	1,813 (19.7%)	794 (20.3%)	
T4	87 (0.66%)	61 (0.66%)	26 (0.67%)	
N				0.295
N0	11,983 (91.4%)	8,430 (91.6%)	3,553 (91.0%)	
N1	1,122 (8.56%)	772 (8.39%)	350 (8.97%)	
M				0.724
M0	10,665 (81.4%)	7,481 (81.3%)	3,184 (81.6%)	
M1	2,440 (18.6%)	1,721 (18.7%)	719 (18.4%)	
Tumor size				0.963
<40 mm	6,109 (46.6%)	4,284 (46.6%)	1,825 (46.8%)	
41–80 mm	4,680 (35.7%)	3,293 (35.8%)	1,387 (35.5%)	
>80 mm	2,316 (17.7%)	1,625 (17.7%)	691 (17.7%)	
Surgery				0.125
No	5,796 (44.2%)	4,110 (44.7%)	1,686 (43.2%)	
Local tumor excision	1,269 (9.68%)	911 (9.90%)	358 (9.17%)	
Partial nephrectomy	1,519 (11.6%)	1,048 (11.4%)	471 (12.1%)	
Radical nephrectomy	4,521 (34.5%)	3,133 (34.0%)	1,388 (35.6%)	
Chemotherapy				1.000
No/Unknown	12,020 (91.7%)	8,440 (91.7%)	3,580 (91.7%)	
Yes	1,085 (8.28%)	762 (8.28%)	323 (8.28%)	
Radiation				0.757
No/Unknown	12,467 (95.1%)	8,758 (95.2%)	3,709 (95.0%)	
Yes	638 (4.87%)	444 (4.83%)	194 (4.97%)	

### Univariate and Multivariate Cox Regression Analysis

We analyzed patient prognostic factors using univariate and multivariable Cox regression models. The univariate Cox regression model showed that age, year of diagnosis, race, marriage, histological grade, tumor size, TNM stage, surgery, radiotherapy, and chemotherapy influenced patients' CSS. Multivariate Cox regression analysis showed that age, histological grade, TNM stage, tumor size, surgery, radiotherapy and chemotherapy were prognostic factors affecting patients' CSS. Cox regression analysis results are shown in Table 2.

**Table 2:** Proportional subdistribution hazard analyses of CSS in training cohort.

	**CSS**		
	**HR**	**95%CI**	**P**
Age			
65–74			
75–84	1.20	1.09–1.32	<0.001
≥85	1.50	1.32–1.7	<0.001
Race			
White			
Black	0.94	0.81–1.08	0.35
Other	0.89	0.75–1.06	0.18
Sex			
Male			
Female	0.87	0.79–0.95	0.001
Marital			
Married			
Unmarried or Domestic Partner/Single	1.05	0.93–1.19	0.4
Separated/Divorced/ Widowed	1.10	1–1.21	0.56
Year of diagnosis			
2004–2010			
2010–2018	0.88	0.81–0.95	0.002
Laterality			
Left			
Right	1.08	1–1.17	0.057
Grade			
I			
II	0.95	0.73–1.23	0.7
III	1.37	1.06–1.78	0.017
V	1.76	1.31–2.37	<0.001
Unknown	1.19	0.92–1.52	0.18
T			
T1a			
T1b	1.56	1.19–2.04	0.001
T2	2.00	1.54–2.6	<0.001
T3	2.38	1.87–3.04	<0.001
T4	2.03	1.24–3.32	0.005
N			
N0			
N1	1.49	1.32–1.68	<0.001
M			
M0			
M1	4.32	3.84–4.87	<0.001
Tumor size			
<40 mm			
41–80 mm	1.26	0.99–1.59	0.06
>80 mm	1.44	1.13–1.82	0.003
Surgery			
No			
Local tumor excision	0.47	0.37–0.58	<0.001
Partial nephrectomy	0.30	0.24–0.39	<0.001
Radical nephrectomy	0.49	0.42–0.56	<0.001
Chemotherapy			
No/Unknown			
Yes	0.99	0.88–1.12	0.92
Radiation			
No/Unknown			
Yes	1.20	1.04–1.38	0.013

### Nomogram Construction for 1, 3, and 5-Year CSS

**Figure 2:**
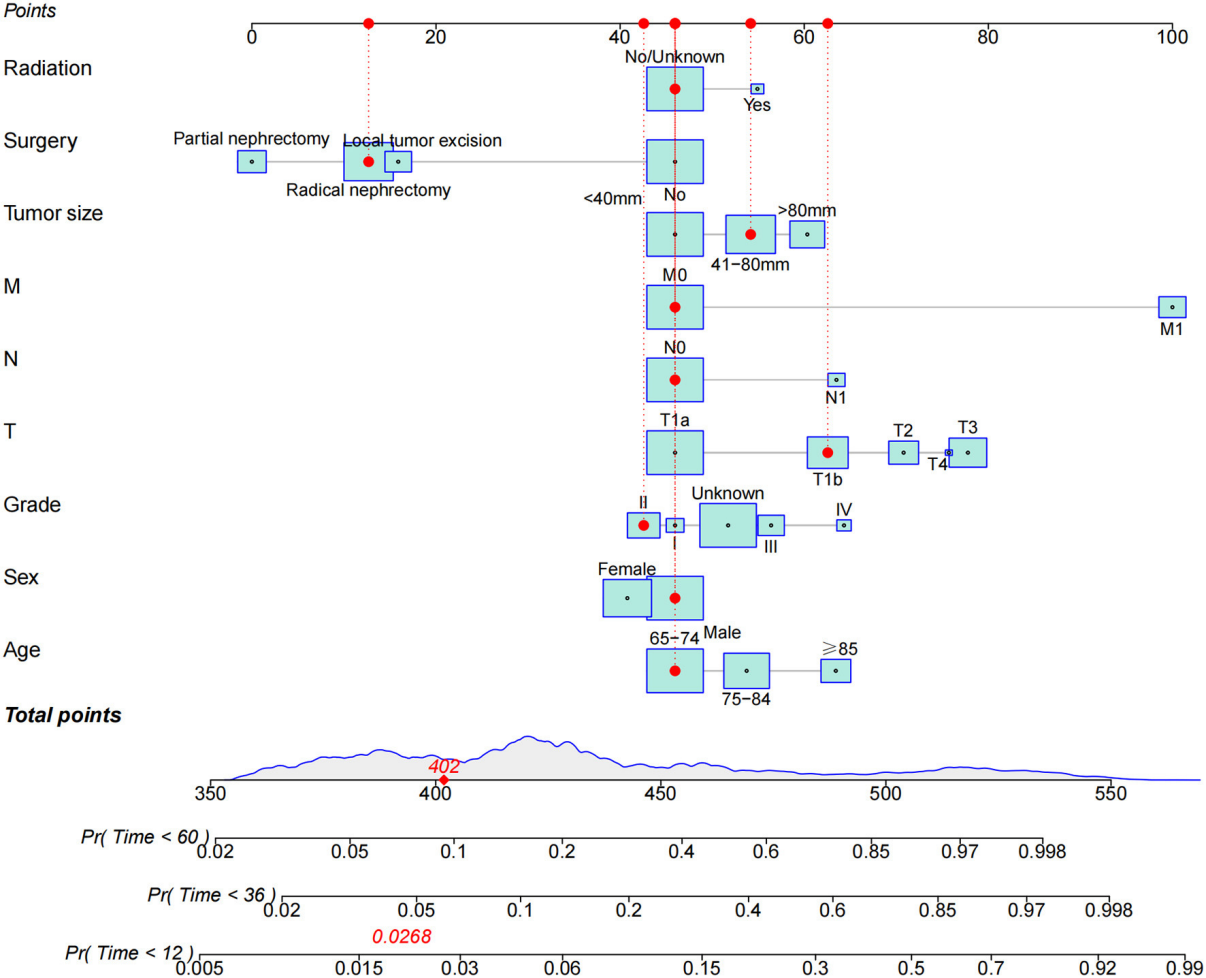
The nomogram for predicting 1-, 3-, and 5-year CSS in elderly patients with pRCC.

The essence of the nomogram is to visualize the multivariate Cox regression analysis. Therefore, we constructed a nomogram based on multivariate Cox regression analysis to predict CSS in elderly patients with pRCC (Figure 2). As shown in the figure, tumor size and TNM stage are the biggest factors affecting the prognosis of patients, followed by surgery, radiotherapy and chemotherapy. In addition, age and histological grade are also important factors. The larger the tumor, the higher the risk of death, and the higher the TNM stage, the higher the risk of death. Patients with partial nephrectomy had the lowest risk, and patients without surgery had the highest risk. In addition, the older the patient, the higher the risk of death.

### Validation of the Nomogram

**Figure 3:**
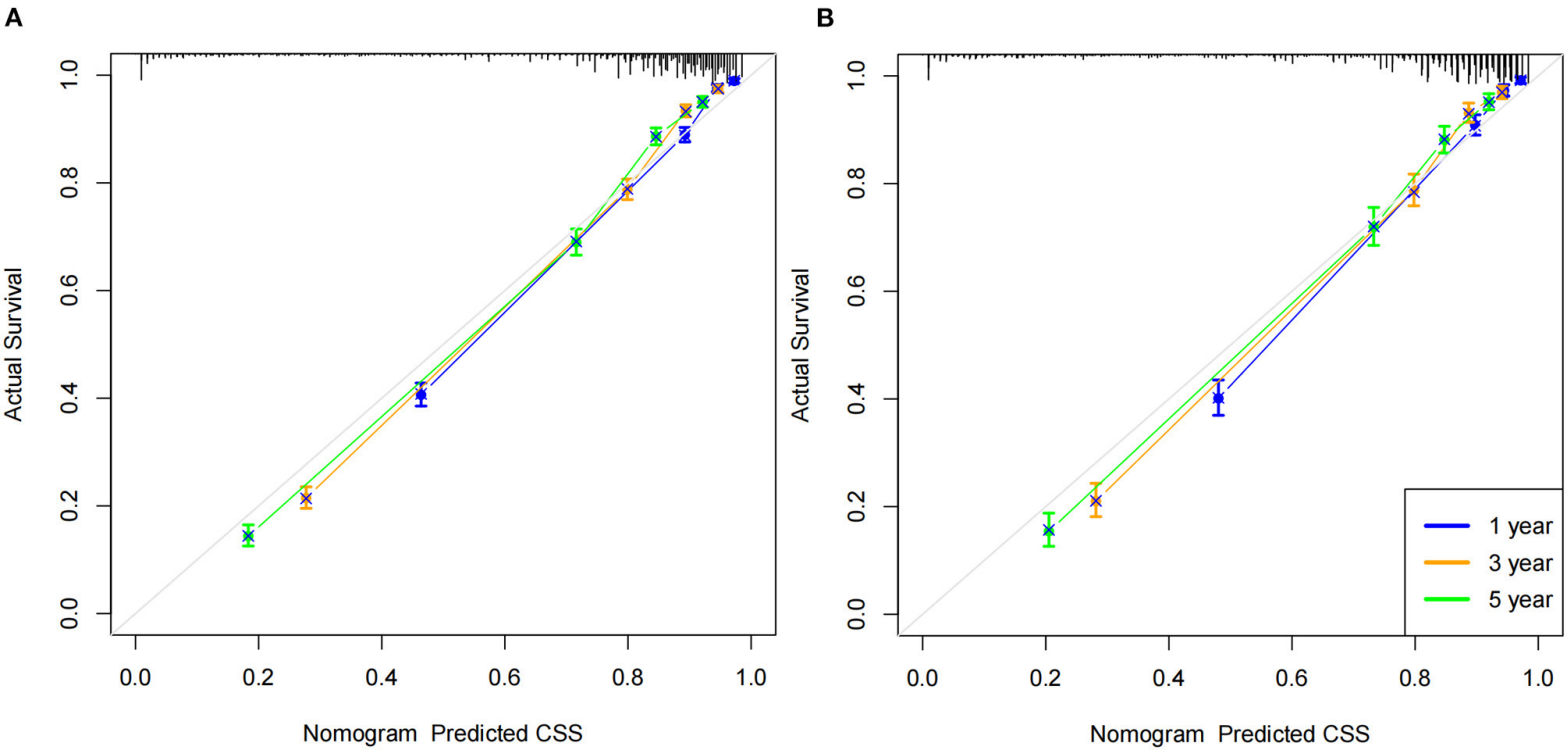
Calibration curve of the nomogram. **(A)** Calibration curves of 1 -, 3 - and 5-year CSS in the training cohort; **(B)** calibration curves of 1-, 3-, and 5-year CSS in the validation cohort.

**Figure 4:**
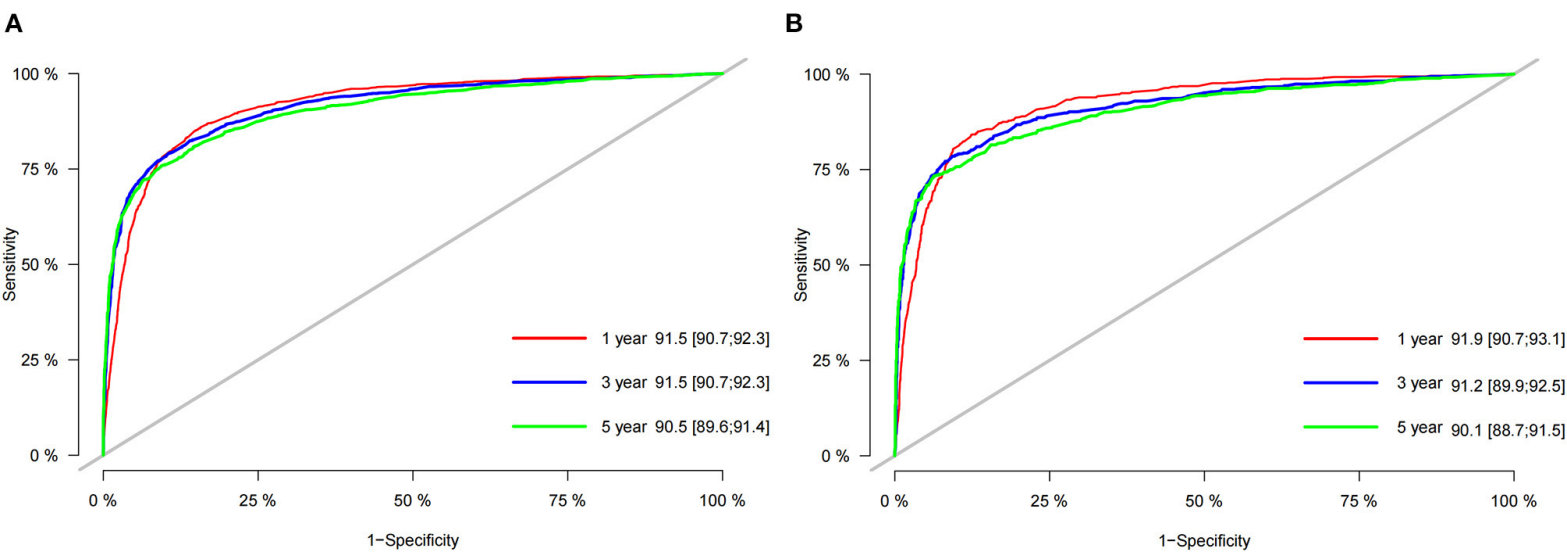
AUC for predicting 1-, 3-, and 5-year CSS in the training cohort **(A)** and the validation cohort **(B)**.

We first use the C-index to validate the discrimination of the prediction model. In the training cohort and validation cohort, the C-index was 0.853 (95%CI: 0.859–0.847) and 0.855 (95%CI: 0.865–0.845), respectively. The results showed that the nomogram had good discrimination. The calibration curve was also used to validate the accuracy of the model. The calibration curve showed that the predicted value of the nomogram was highly consistent with the actual observed value, indicating that the prediction model had good accuracy (Figure 3). In the training cohort, the nomogram' 1-, 3- and 5-year AUC values were 91.5, 91.5 and 90.2, respectively. In the validation cohort, the nomogram' 1-, 3- and 5-year AUC values were 92.1, 91.2 and 90.3, respectively. It shows that the nomogram has good discrimination (Figure 4).

### Clinical Application of the Nomogram

**Figure 5:**
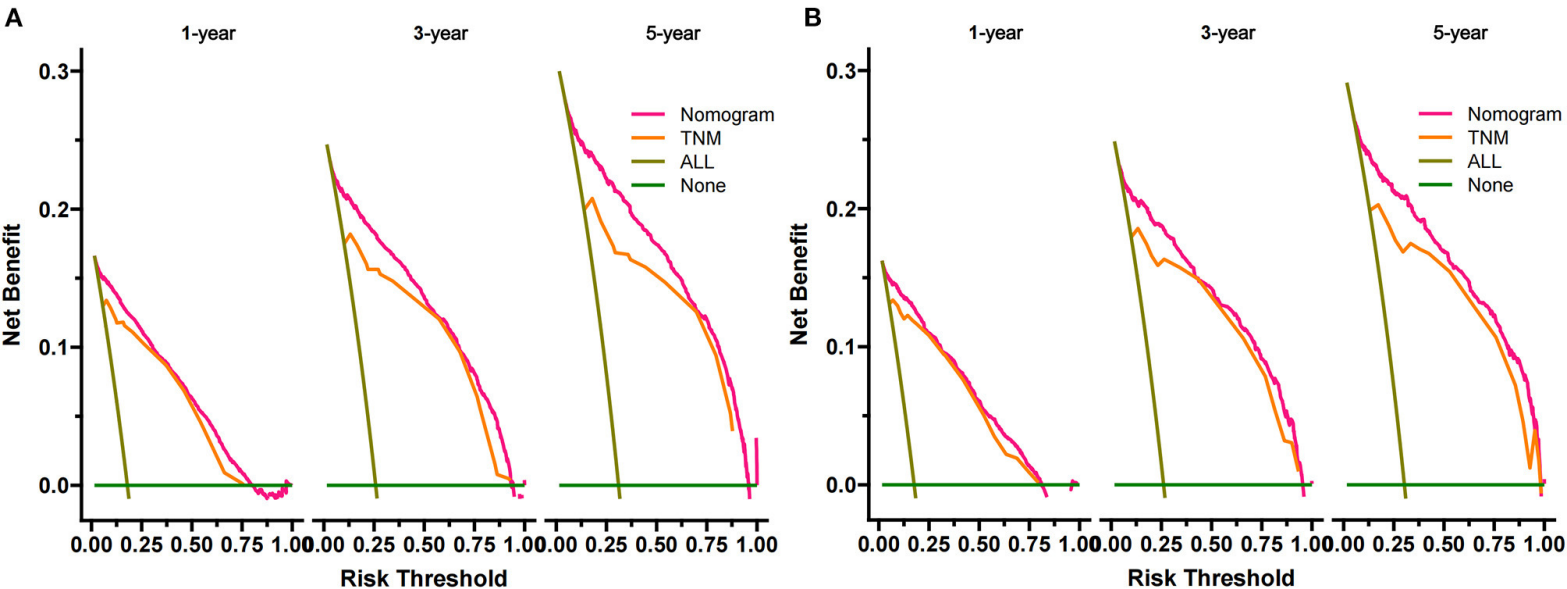
DCA of the nomogram in the training cohort **(A)** and the validation cohort **(B)**. The Y-axis represents a net benefit, and the X-axis represents threshold probability. The green line means no patients died, and the dark green line means all patients died. When the threshold probability is between 0 and 100%, the net benefit of the model exceeds all deaths or none.

**Figure 6:**
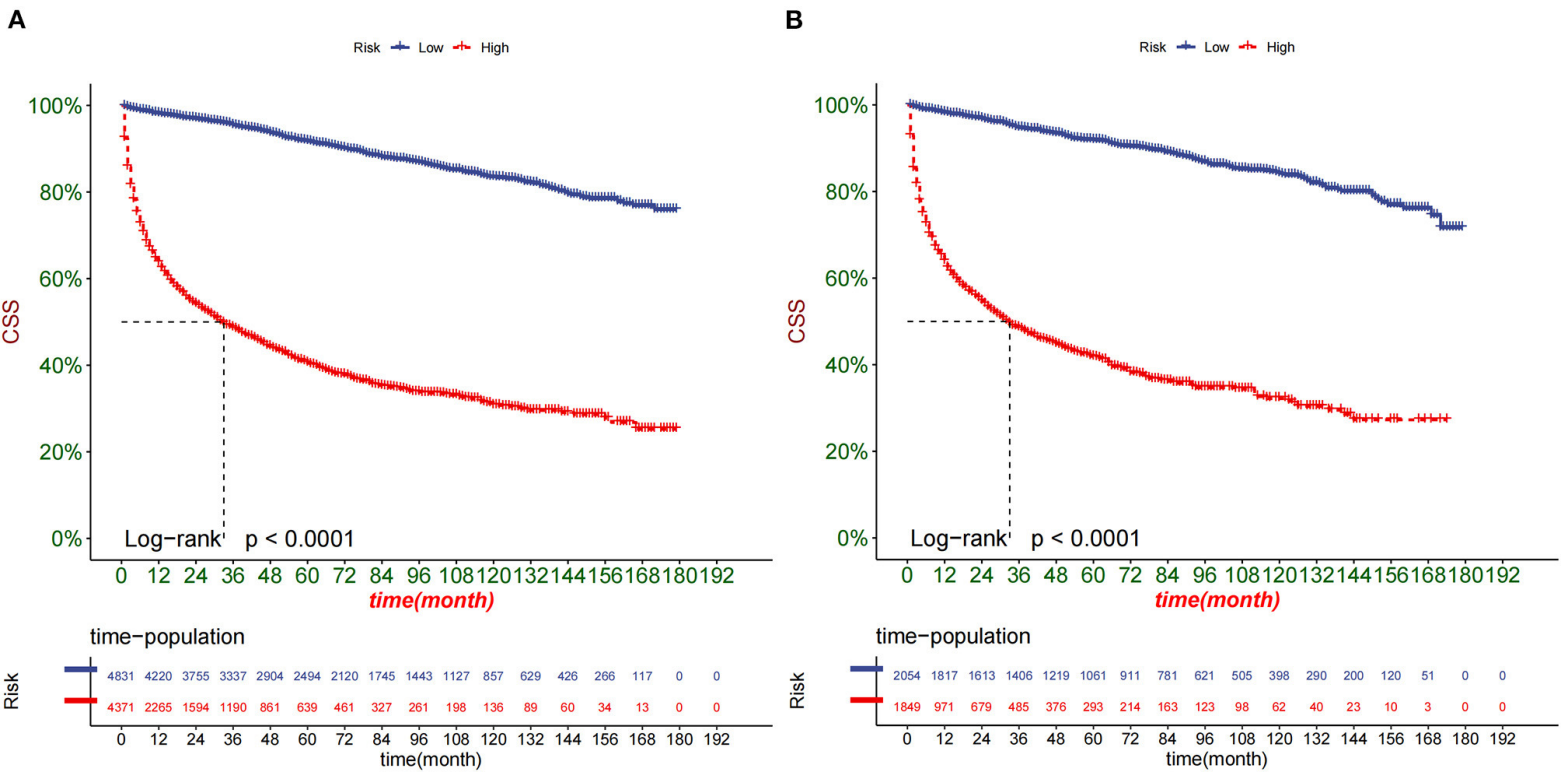
Kaplan-Meier curves of patients in the low-risk and high-risk groups in the training cohort **(A)** and the validation cohort **(B)**.

**Figure 7:**
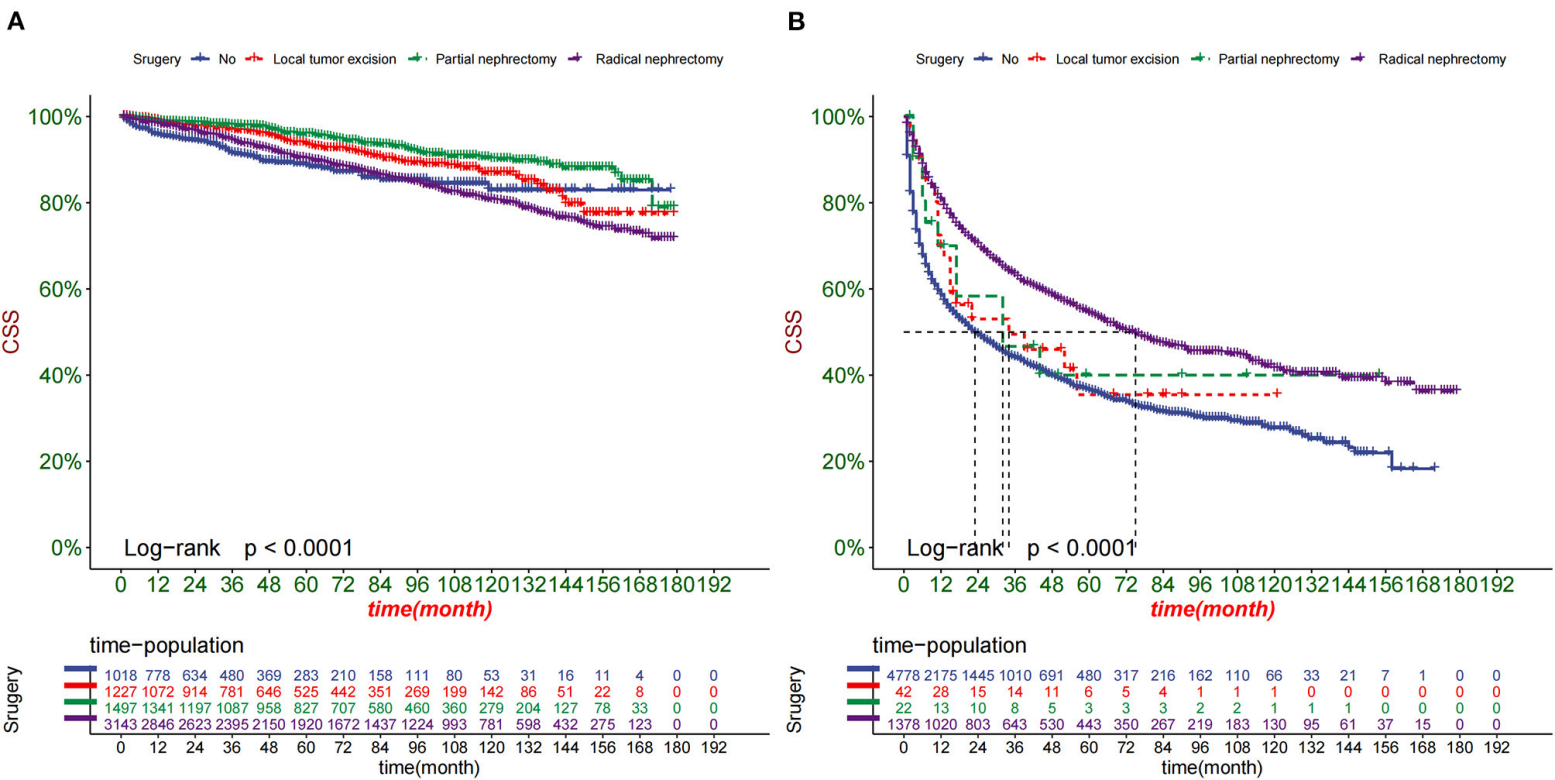
Kaplan-Meier curves of patients with different surgery in the low-risk group **(A)** and high-risk group **(B)**.

DCA was used to test the clinical application value of the prediction model. DCA showed that the nomogram had potential clinical application value and was more practical than the traditional TNM staging (Figure 5). Based on the nomogram, we calculated the risk values of all patients and divided them into the high-risk group using ROC cut-off values (total score > 95.7) and the low-risk group (total score ≤95.7). The K-M curve showed that the survival rate of patients in the high-risk group was significantly lower than that in the low-risk group (Figure 6). In the high-risk group, 1-, 3-, and 5-year survival rates were 64.7, 47.9, and 42.2%, respectively. In the low-risk group, 1-, 3-, and 5-year survival rates were 98.4, 95.7, and 92.2%, respectively. In addition, we analyzed surgical procedures in the high-risk and low-risk groups. In the low-risk group, survival was highest in patients who received partial nephrectomy and lowest in radical nephrectomy. In the high-risk group, survival was highest who underwent radical nephrectomy and lowest for those who did not (Figure 7).

## Discussion

RCC accounts for about 2% of all cancer diagnoses and deaths worldwide, with higher rates in developed countries. RCC is the most common type of renal malignancy, accounting for more than 90%. pRCC accounts for 10–20% of all renal cell carcinomas. However, compared with other types of RCC, pRCC lacks specific clinical manifestations and associated symptoms, and more importantly, pRCC does not have typical radiographic findings. In addition, some elderly patients may present with perirenal abscesses due to weakened immunity. It brings great difficulties to the diagnosis and treatment of pRCC for clinicians (15). According to recent reports, the overall prognosis of pRCC is slightly better than that of clear cell renal carcinoma and chromophobe renal carcinoma (16). However, in clinical practice, in addition to TNM staging, there is currently a lack of a model that can accurately predict the prognosis of elderly patients with pRCC.

Nomogram is a data-based graphical computing tool that can estimate the risk of a disease based on staging systems such as the American Joint Commission on Cancer (AJCC) and other key risk factors related to prognosis (17). Compared with traditional TMN staging, nomogram has better accuracy in prognostic prediction and can provide better advice and help for clinicians in diagnosis and treatment (18). To our knowledge, there have been no reports on the prognosis of elderly patients with pRCC. In addition, due to the relatively low incidence of pRCC, it is difficult to collect a large sample size for single-center studies of this disease to draw reliable conclusions (19). Therefore, it is particularly important to establish a more reliable and accurate predictive model for pRCC in the elderly. This study collected data from the SEER Database, a large sample database established in 1973. At present, the database covers 18 countries and regions, effectively avoiding the lack of sample size and single type (20).

In this study, we established and validated a new nomogram to accurately predict CSS in elderly pRCC. Previous studies have found that pRCC has a higher incidence and worse survival rate in elderly patients (21). Our study also confirmed that age is a key factor in the development of pRCC in the elderly. As we age, it is well known that the risk of genetic mutations leading to cancer increases. Studies have shown that age plays a key role in the survival rate of various cancers (22, 23). Huang et al. found by propensity matching comparison that pRCC had a significantly worse prognosis than ccRCC in patients aged ≤ 45 years (24). Su et al. collected the SEER database of pRCC patients who underwent nephrectomy from 2010 to 2016 for analysis. They confirmed that age is a key factor influencing the all-cause mortality of pRCC (25). The study of Nelson et al. also found that the survival rate of mRCC patients aged ≥75 years was significantly lower than that of patients aged <75 years (26). There is no consensus on defining the age of elderly patients, but more than 60% of initial cancer diagnoses and more than 70% of cancer deaths occur in patients over 65 years old (8). To improve the accuracy and representativeness of the prediction model, pRCC patients over 65 years old were included in this study.

At the same time, we found that tumor size is a major risk factor affecting the prognosis of pRCC in the elderly, and larger tumor occurrence often suggests poor prognosis, which is consistent with the results of previous studies. Hutterer et al. previously established a nomogram to predict the survival rate of RCC and found that tumor size was an important risk factor (27). Zastrow et al. also found that tumor size was a risk factor for the long-term survival of pRCC (28).

As is known to all, the TNM staging system is a common method for clinical evaluation of various malignant tumors, which helps to judge the prognosis of cancer patients and guide clinicians to take better treatment (29, 30). However, only the size of the tumor, the presence of lymph node metastasis, and distant metastasis were used as criteria. Age, marital status, surgical method, chemotherapy and radiotherapy, and other important factors that have been proven to affect cancer patients' overall survival rate (OS) were ignored (31). Our study found that in elderly patients undergoing pRCC surgery, partial nephrectomy (PN) had the best prognosis, radical nephrectomy (RN) was intermediate, and local tumor resection had the worst prognosis. It is consistent with most research conclusions. Shum et al. showed that in T2 stage malignancies, the OS of PN was significantly better than that of RN (32). Hellenthal et al. collected RCC patients from 1988 to 2005 in the SEER database. After analysis, it was concluded that PN could still significantly improve OS even with tumor metastasis, benefiting mRCC patients (33). In recent years, postoperative radiotherapy has been gradually included in various cancer guidelines because of its good effect as a key means of postoperative treatment. RCC is sensitive to radiotherapy, and the strategy has been agreed upon.

Interestingly, we found that postoperative chemotherapy did not improve CSS in elderly patients with pRCC, which is consistent with Tachibana and De Vries-Brilland et al. The former retrospectively analyzed RCC patients who received nivolumab and ipilimumab as a first-line treatment between December 2015 and May 2020 and found that the chemotherapy regimen achieved good results in ccRCC, but intermediate results in pRCC (34). The latter summarized the treatment methods of pRCC and concluded that the existing chemotherapy regimens were not sensitive to pRCC. The combination of immune checkpoint inhibitors (ICI) and tyrosine kinase inhibitors (MET) may be a new direction for the treatment of pRCC in the future (35).

Finally, the newly constructed nomogram model for predicting CSS in elderly patients with pRCC includes many factors, such as diagnosis age, tumor size, TNM grade, Fuhrman grade, and operation at the primary site, which is convenient for clinical information collection. In summary, the nomograms we developed can accurately predict CSS at 1, 3, and 5 years in patients with pRCC. Furthermore, we used AUC, C-index, and DCA to validate its accuracy and predictive power for elderly papillary renal cell carcinoma.

However, there are still some limitations in this study. First of all, the SEER database does not include BMI, smoking, alcohol consumption, etc. These are important factors affecting patients' survival. However, we included the basic patient information cohort, tumor information, and other key factors. Secondly, because this study is retrospective, there is inevitable selection bias. Finally, the prediction model is only validated internally, and further external validation is necessary to validate the model's accuracy.

## Conclusion

In this study, we explored the prognostic factors of elderly pRCC patients and the patient's age, histological grade, TNM stage, tumor size, surgery, radiotherapy, and chemotherapy as independent risk factors affecting patients CSS. We constructed a nomogram to predict the CSS of elderly pRCC patients with good accuracy and reliability, which can help doctors and patients make clinical decisions.

## Data Availability Statement

Publicly available datasets were analyzed in this study. This data can be found here: https://seer.Cancer.gov/.

## Ethics Statement

The data of this study is obtained from the SEER database. The patients' data is public, so this study does not require ethical approval and informed consent.

## Author Contributions

JW and CZ designed the study. CZ, JW, LL, YX, and HT collected and analyzed the data. JW drafted the initial manuscript. CZ, KZ, and BY revised the article critically. CZ, ZY, and BY reviewed and edited the article. All authors approved the final manuscript.

## Funding

This study was supported by Yunnan Education Department of Science Research Fund (No. 2020 J0228), Kunming City Health Science and Technology Talent “1000” Training Project (No. 2020- SW (Reserve)-112), Kunming Health and Health Commission Health Research Project (No. 2020-0201-001), and Kunming Medical Joint Project of Yunnan Science and Technology Department (No. 202001 AY070001-271). The funding bodies played no role in the study's design and collection, analysis and interpretation of data, and writing the manuscript.

## Conflict of Interest

The authors declare that the research was conducted in the absence of any commercial or financial relationships that could be construed as a potential conflict of interest.

## Publisher's Note

All claims expressed in this article are solely those of the authors and do not necessarily represent those of their affiliated organizations, or those of the publisher, the editors and the reviewers. Any product that may be evaluated in this article, or claim that may be made by its manufacturer, is not guaranteed or endorsed by the publisher.
